# Fatalities Involving Khat in Jazan, Saudi Arabia, 2018 to 2021

**DOI:** 10.3390/toxics11060506

**Published:** 2023-06-04

**Authors:** Ghassan Shaikhain, Mohammed Gaballah, Ahmad Alhazmi, Ibrahim Khardali, Ahmad Hakami, Magbool Oraiby, Sultan Alharbi, Mohammad Tobaigi, Mohammed Ghalibi, Mohsen Fageeh, Mohammed Albeishy, Ibraheem Attafi

**Affiliations:** 1 Forensic Toxicology Services, Forensic Medical Center, Ministry of Health, Jazan 45142, Saudi Arabia; 2 Forensic Medicine Services, Forensic Medical Center, Ministry of Health, Jazan 45142, Saudi Arabia

**Keywords:** forensic toxicology, khat, cathinone, cathine, postmortem

## Abstract

Interpreting fatalities involving khat is challenging due to a lack of data on cathinone and cathine reference concentrations in postmortem tissues. This study investigated the autopsy findings and toxicological results of fatalities involving khat in Saudi Arabia’s Jazan region from 1 January 2018 to 31 December 2021. All confirmed cathine and cathinone results in postmortem blood, urine, brain, liver, kidney, and stomach samples were recorded and analyzed. Autopsy findings and the manner and cause of death of the deceased were assessed. Saudi Arabia’s Forensic Medicine Center investigated 651 fatality cases over four years. Thirty postmortem samples were positive for khat’s active constituents, cathinone and cathine. The percentage of fatalities involving khat was 3% in 2018 and 2019 and increased from 4% in 2020 to 9% in 2021, when compared with all fatal cases. They were all males ranging in age from 23 to 45. Firearm injuries (10 cases), hanging (7 cases), road traffic accident (2 cases), head injury (2 cases), stab wounds (2 cases), poisoning (2 cases), unknown (2 cases), ischemic heart disease (1 case), brain tumor (1 case), and choking (1 case) were responsible for the deaths. In total, 57% of the postmortem samples tested positive for khat only, while 43% tested positive for khat with other drugs. Amphetamine is the drug most frequently involved. The average cathinone and cathine concentrations were 85 and 486 ng/mL in the blood, 69 and 682 ng/mL in the brain, 64 and 635 ng/mL in the liver, and 43 and 758 ng/mL in the kidneys, respectively. The 10th–90th percentiles of blood concentrations of cathinone and cathine were 18–218 ng/mL and 222–843 ng/mL, respectively. These findings show that 90% of fatalities involving khat had cathinone concentrations greater than 18 ng/mL and cathine concentrations greater than 222 ng/mL. According to the cause of death, homicide was the most common fatality involving khat alone (77%). More research is required, especially toxicological and autopsy findings, to determine the involvement of khat in crimes and fatalities. This study may help forensic scientists and toxicologists investigate fatalities involving khat.

## 1. Introduction

Khat is the common name for the plant known by its scientific name as Catha edulis (Vahl) Forssk. ex Endl. Typically, its leaves are chewed to produce stimulant effects such as alertness, euphoria, and increased motor activity [1]. The two major active components of khat are cathine and cathinone. They are listed in the controlled substances act as psychotropic substances Schedules III and I, respectively, and the khat plant is listed as a controlled plant [2]. Despite the low concentrations of cathine and cathinone in khat leaves (which range from 0.1% to 0.2% and from 0.1% to 0.3%, respectively), long-term consumption of large amounts of khat leaves can cause toxicity [3,4]. In addition to those compounds, khat leaves also contain sterols, tannins, cathedulins (polyhydroxylated sesquiterpenes), triterpenes, and flavonoids [5].

The most potentially toxic effects of khat consumption are hyperthermia, insomnia, anorexia, constipation, urinary retention, hypertension, myocardial infarction, and arrhythmia [6,7,8,9]. Hepatitis was also reported in two khat users, which was resolved by discontinuing khat use, but relapse occurred when khat use was resumed in both cases [10]. There are also reported cases of cerebral hemorrhage, psychoactive disorders, and cancer [5,11]. Chewers of khat typically consume 50–200 g of fresh khat leaves per day [12]. This amount of khat leaves consumed daily induces psychological dependence and tolerance, leading to an increased daily consumption [13,14,15]. The metabolic cytochrome P450 enzymes 3A4, 2D6, 2C19, and 1A2 are affected by khat consumption [16,17]. Hence, the inhibitory effects of khat on metabolic enzymes may alter plasma concentrations, which may impact the efficacy and safety of drug therapy.

Both cathine and cathinone are basic compounds with pKa values of 9.37 and 7.55, respectively, and are susceptible to postmortem redistribution [18]. As a parent drug, cathine and cathinone are excreted in human urine. Furthermore, they each have a minor metabolite, pseudoephedrine and diethylpropion [19,20]. Cathinone was also rapidly converted to norephedrine and nor-pseudoephedrine [21]. Consequently, the aforementioned metabolites may indicate Khat consumption [22].

The toxicity of khat exposure has been demonstrated by previous studies, and the possibility of lethality cannot be ruled out. To rule out an overdose as the cause of death, toxicologists must evaluate and interpret the substance concentrations present in postmortem samples. Substance concentrations must be quantified, reported, and published to expand the limited published evidence on toxicological findings for fatalities involving khat. The autopsy findings and other circumstances surrounding death are also important indicators of substance-related deaths. The results of an autopsy finding will also be important for figuring out what concentrations of cathine and cathinone are dangerous and which are fatal. Studying the prevalence of fatalities involving khat is also important for prevention, treatment, and education. The purpose of this study were (a) to analyze fatalities involving khat in Jazan, Saudi Arabia, and (b) to explore the disposition of cathine and cathinone in postmortem tissues.

## 2. Materials and Methods

### 2.1. Study Design and Data Collection

All fatal forensic cases received at the Poison Control and Medical Forensic Chemistry Center in Jazan, Saudi Arabia, from the 1st of January 2018 to the 31st of December 2021 were retrospectively evaluated. The data were collected from the OTARR electronic system using the data collection form. All acquired data regarding the toxicological study results and accident summary, including the manner of death in the forensic cases involving khat, were analyzed. These data included forensic cases that are confirmed as positive for the presence of cathine and cathinone and excluded the forensic cases that are negative for the presence of cathine and cathinone.

All information regarding the autopsy finding, manner, and causes of death in fatalities involving khat within the period of study was revised, defined, and recorded by the medical forensic examiner experts at the Jazan Forensic Medicine Center. The autopsy finding data in the autopsied cases were clearly defined and tabulated. The manner of death was classified as follows: suicidal, homicidal, accidental, and undetermined. The identification of hazards for cathine and cathinone, as well as forensic cases involving their exposure, have been evaluated.

### 2.2. Toxicological Analysis

Drugs of abuse including amphetamines, cocaine, cannabinoids, opiates, barbiturates, and benzodiazepines in all autopsied samples are primarily screened via immunoassay analysis using the RANDOX system (Evidence Plus; Randox Laboratories, Crumlin, UK). Volatiles including methanol, ethanol, isopropanol, and acetone are routinely analyzed using headspace-gas chromatography. Using liquid chromatography–mass spectrometry, the immunoassay positive results for amphetamine-type stimulants (including amphetamine, methamphetamine, cathine, and cathinone) were confirmed and quantified, while gas chromatography–mass spectrometry was used to exclude other drugs.

Cathine and cathinone concentrations were identified and quantified via the liquid chromatography–ion trap mass spectrometry (LCQ Fleet Ion Trap LC/MS) method (Thermo Fisher Scientific, Waltham, MA, USA). For sample preparation, one gram of each organ tissue was homogenized in one milliliter of deionized water and then centrifuged for 15 min at 3000× *g* using a Heraeus Labofuge 400 centrifuge (Thermo Fisher Scientific, Waltham, MA, USA). The supernatant was then mixed with 1 mL of phosphate buffer pH 6 and vortexed for solid phase extraction (SPE) using DAU extraction columns (UCT, Bristol, PA, USA). Prior to the extraction procedure, one milliliter of each sample’s homogenate was spiked with 20 μL of a prepared stock solution of 3,4-methylenedioxymethamphetamine (MDMA) at a concentration of 50 μg/mL as an internal standard. SPE columns were preconditioned with 3 mL methanol and 3 mL deionized water and equilibrated with 1 mL phosphate buffer at pH 6. Upon sample load completion, SPE columns were washed with 3 mL of deionized water, 1 mL of 0.1 M acetic acid, and 3 mL of methanol and dried for 10 min under a nitrogen stream. Next, cathine, cathinone, and MDMA were eluted into 12 mL glass tubes with 3 mL of a mixture of dichloromethane, isopropanol, and ammonium hydroxide (78:20:2, *v*:*v*:*v*). Then, one drop of 0.1 M hydrochloric acid (HCl) was added into each tube, and all elutions were evaporated to dryness under nitrogen stream. Finally, all samples were reconstituted with 100 μL of the aqueous proportion of the mobile phase (10 mM ammonium formate with 0.11% formic acid) for LCQ Fleet Ion Trap LC/MS analysis.

Calibration samples were prepared by spiking 1 mL of blank tissue homogenates with cathine, cathinone, and MDMA (Lipomed, Arlesheim, Switzerland). Six calibration points were prepared at concentrations of 50, 100, 250, 500, 1000, 2000, 3000, 4000, and 6000 ng/mL of cathine and cathinone and 1000 ng/mL MDMA as the internal standard. Postmortem samples were diluted prior to extraction and reanalyzed when concentrations exceeded the upper limits of quantification. Three quality control samples were prepared by spiking 1 mL of the blank homogenate tissues with cathine and cathinone at the concentrations of 100, 250, and 500 ng/mL and the concentration of 1000 ng/mL of MDMA as an internal standard.

An LCQ Fleet Ion Trap LC/MS system (Thermo Fisher Scientific, Waltham, MA, USA), employing an LCQ fleet mass analyzer coupled with a Surveyor Auto-Sampler and a Surveyor Quaternary Pump and managed by X-Caliber Software (Themo Scientific, USA), was used. Briefly, 10 μL of each sample was injected by an autosampler. The chromatographic separation of cathine, cathinone, and MDMA was achieved with a HPLC column (Hypersil GOLD, 5 μm, 150 × 4.6 mm, Thermo Fisher Scientific, Waltham, MA, USA), using mobile phase A (ammonium formate (10 Mm; 0.639 mg ammonium formate in 1 L HPLC water) and mobile phase B ( formic acid in acetonitrile (0.1%; 1 mL formic acid in 999 mL acetonitrile). Gradient elution was performed as follows: 0–1 min, 100% A; and 1–7.5 min, 80% A; 7.5–8.5 min, 50% A, 8.5–9.5 min, 0% A; 9.5–10.5 min, 50% A; and 10.5–11.5 min 100% A. Flow rate was 300 μL/min, and the injection volume was 5 μL. The electrospray ion source (ESI), as an optimized tuning profile of ATS, runs in positive ionization mode with 5 kV spraying voltage, 275 °C capillary temperature, and sheath gas value 30. The mass analyzer runs in the scan mode, scanning at *m*/*z* 152 for cathine, *m*/*z* 150 for cathinone, and *m*/*z* 194 for MDMA. Cathine, cathinone, and MDMA are fragmented in the collision cell with helium gas in the Pulsed q collision-induced dissociation (PQD) mode into *m*/*z* 134 and 117 for cathine, *m*/*z* 132 and 105 for cathinone, and *m*/*z* 163 and 135 for MDMA. The PQD values for cathine and cathinone were 19 and 22 for MDMA. Qualitative and quantitative analyses were performed using X-Caliber Software. The analytical method employed in this study was developed and validated in our laboratory, and it is already routinely used to analyze amphetamine and related substances, with in-house modifications [23].

The general unknown screening analysis was performed via gas chromatography–mass spectrometry (GC/MS) analysis (GC/MS Agilent Technologies, Santa Clara, CA, USA). Each sample (one ml of a homogenate sample) was mixed with 1 mL of the phosphate buffer (pH 6). One milliliter of the homogenate sample was extracted via the solid phase extraction (SPE) technique using DAU extraction columns (UCT, Bristol, PA, USA) according the manufacture’s instructions. For instance, columns were conditioned with 3 mL of methanol and then 3 mL of deionized water and equilibrated with 1 mL of the phosphate buffer (pH 6). Two milliliters of each sample was loaded and allowed to pass slowly. Then, the columns were washed with 3 mL of deionized water followed by 1 mL of 0.1 M acetic acid and allowed to dry for 15 min under a flow of air. The first elution was collected by adding 2 mL of ethyl acetate/hexane (50:50, *v*:*v*). Thereafter, the columns were washed with 3 mL of methanol, 2 mL of the second elution (dichloromethane/isopropanol/ammonium hydroxide; 78:20:2, *v*:*v*:*v*) was added, and the sample was dried under nitrogen. All samples were reconstituted with methanol (100 μL), then vortexed, and transferred to GC/MS autosampler vials for GC/MS analysis.

The Thermo Fisher Scientific (TR-5MS) separation column had the following properties: 30 m length, internal diameter (ID) 0.25 mm, and film thickness 0.25 μm. Helium was used as the carrier gas with 1 mL/min flow rate. A total of 2 μL of each sample was injected into the splitless mode at an injection port with a temperature of 260 °C. The GC thermal program started at 80 °C and lasted 1.5 min. The thermal program then increased the temperature at the initial ramp to 210 °C at a rate of 30 °C/min and then slowed to 20 °C/min to reach the final temperature of 320 °C, which was held for 11 min. Electron ionization (EI) was used as the ion source in MS, and the analysis was carried out in scanning mode with an electron energy of 70 eV. The temperature of the ion source and transfer line was set at 230 °C. The mass spectral libraries from Wiley and the National Institute of Standards and Technology (NIST) were used to identify the GC/MS mass spectra of unknown substances.

### 2.3. Statistical Analysis

All variables were categorized and tabulated using descriptive statistics. Means, standard error of mean (SEM), and median were presented. SigmaPlot for Windows Version 11.0 was used to analyze all of the data.

## 3. Results

The Forensic Medicine Center in Jazan, Saudi Arabia, investigated 651 fatal cases over four-year period. Thirty of the cases had postmortem samples positive for cathinone and cathine, the active ingredients in khat. They were all males ranging in age from 23 to 45. Firearm injuries (10 cases), hanging (7 cases), road traffic accidents (2 cases), head injury (2 cases), stab wounds (2 cases), poisoning (2 cases), unknown (2 cases), ischemic heart disease (1 case), brain tumor (1 case), and choking (1 case) were responsible for the deaths. The toxicological analysis of cathinone and cathine in fatalities involving khat was performed and summarized (Table 1 and Table 2).

The average cathinone and cathine concentrations were 85 and 486 ng/mL in the blood, 1009 and 12,616 ng/mL in the urine, 69 and 682 ng/mL in the brain, 64 and 635 ng/mL in the liver, and 43 and 758 ng/mL in the kidney. The blood concentrations of cathinone and cathine ranged from 18 to 218 ng/mL and from 222 to 843 ng/mL, respectively. In 90% of fatalities involving khat, cathinone concentrations greater than 18 ng/mL and cathine concentrations greater than 222 ng/mL were detected. The retention time for cathine, cathinone and MDMA were 4.4, 4.5, and 5.1 min, respectively. The limits of quantification (LOQ) and detection (LOD) were both 36 ng/mL. Precision and accuracy were within 20% standard deviation and 20% bias, respectively. The extraction recovery ranged from 80 to 90%, with less than 20% carryover. At doses of 100 ng/mL and 6000 ng/mL, the matrix effects (suppression/enhancement) were within the acceptable limits (25%).

**Figure 1:**
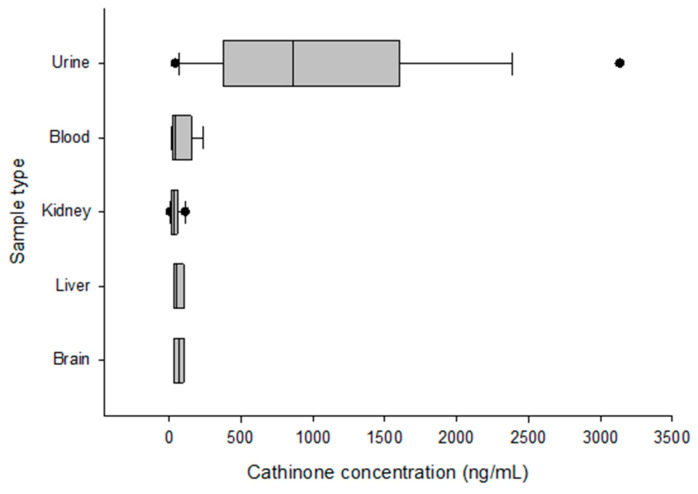
Box plot diagrams of the median and interquartile range of cathinone concentrations detected in fatalities involving khat. Individual dots represent outlier values.

**Figure 2:**
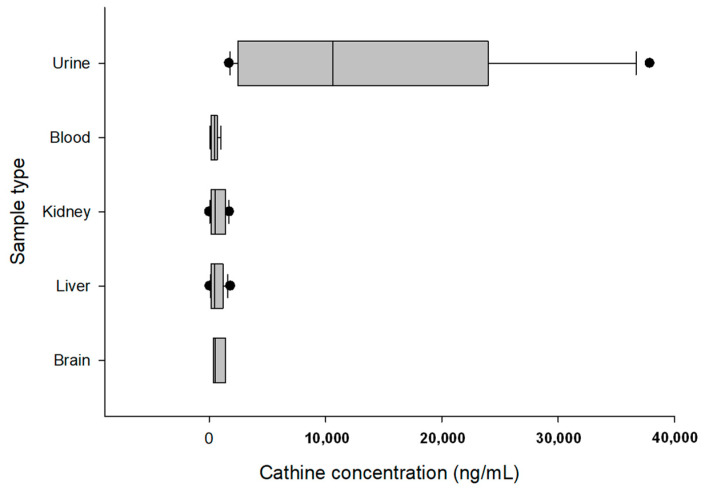
Box plot diagrams of the median and interquartile range of cathine concentrations detected in fatalities involving khat. Individual dots represent outlier values.

The urine samples had the highest 90th percentile concentrations of cathinone (2134 ng/mL) and cathine (36,400 ng/mL). Figure 1 and Figure 2 show box plot diagrams of the median and interquartile range of cathinone and cathine concentrations detected in fatalities involving khat. According to the current study, the number of fatalities involving khat in the last four years increased from 3% in 2018 and 2019 to 4% and 9% in 2020 and 2021, respectively.

Table 3 summarizes the findings of the autopsy. Homicide was the major manner of death (13 of 30 fatalities), and firearm injuries are the leading cause of death among homicide victims (10 of 13 fatalities).

**Figure 3:**
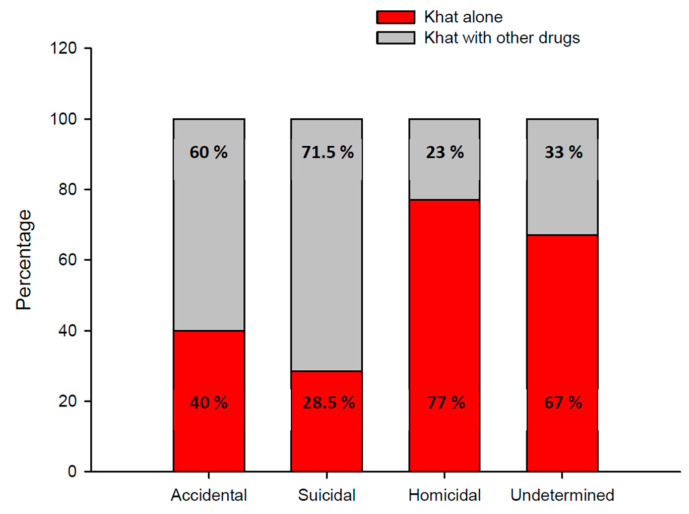
Stacked bar chart of occurrence of the manner of death according to fatalities involving Khat alone or Khat with other drugs.

A stacked bar chart of fatalities involving khat alone or khat in combination with other drugs in different manners of death cases is presented in Figure 3. Homicide occurred more often in fatalities involving khat alone (77%) than in fatalities involving khat in combination with other drugs (23%). Suicide occurred more often in fatalities involving khat in combination with other drugs (71.5%) than in fatalities involving khat alone (28.5%).

**Figure 4:**
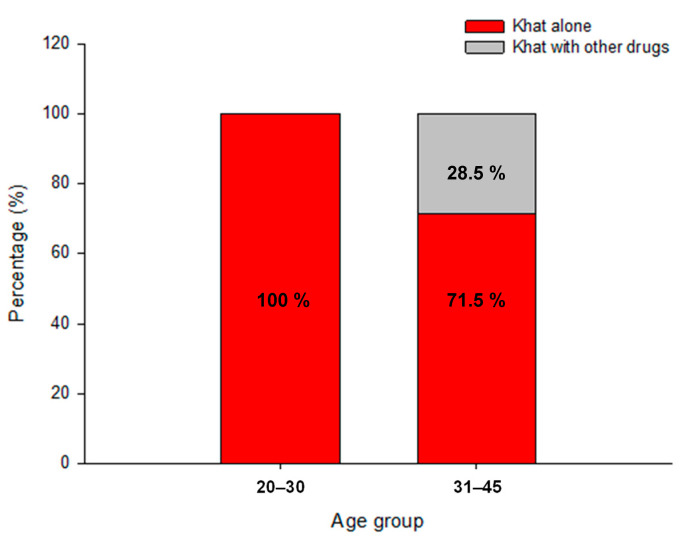
Stacked bar chart of occurrence of the firearm injuries in fatalities involving khat (Khat alone and Khat with other drug) by age group.

Figure 4 is a stacked bar chart showing the occurrence of firearm injuries in fatalities involving khat (khat alone and khat with other drugs) by age group. Remarkably, all fatalities in the 20- to 30-year-old age range and more than 70 percent of fatalities in the 31- to 45-year-old age group were caused by firearm injuries in homicides involving only khat. Another remarkable finding is that five of the seven hanging suicides involved khat in combination with other drugs, and amphetamine is frequently involved in fatalities involving khat (three of seven suicide victims).

## 4. Discussion

The khat plant (Catha edulis (Vahl) Forssk. ex Endl.) is listed by the Saudi Food and Drugs Authority (SFDA) as a prohibited plant under the Act, whereas cathinone and cathine are listed in Schedules I and III, respectively, of the 1971 United Nations Convention on Psychotropic Substances. Khat, on the other hand, is cultivated in Yemen, where it is widely consumed. However, in the Jazan region, which is close to the Yemeni border, 21.4% of students and 28.7% of all surveyed individuals reported that they were currently chewing khat. In Yemen, khat is commonly cultivated and consumed by 68% of the surveyed Yemenis [24,25,26].

In 2020, khat was the most frequently seized plant-based substance, according to the United Nations Office on Drugs and Crime (UNODC, 2022). Khat accounts for 55% of all plant-based substances seized between 2016 and 2020. Saudi Arabia accounted for the most total khat seizures in 2019 [27]. In addition, chewing khat has become increasingly illegal in Europe, not because it is associated with toxicity but because it has fallen into the hands of organized crime networks [28]. There is a significant risk of toxicity for those who consume khat excessively, a problem that is common among the majority of khat users who consume large amounts of khat on a regular basis. According to a recent study, 73.5 percent of people who are dependent on khat chewing have consumed half a bundle or more of khat, and 55.9 percent of those people chew khat more than three times per week for an average of more than six hours each session [29]. Approximately 200 g of khat leaves are contained within each bundle.

Few articles have documented fatalities associated with khat, and there is currently no established reference range for cathinone and cathine that is toxic and lethal [22,30,31]. Cathinone and cathine were found in the blood, urine, vitreous humor, brain, liver, kidney, and stomach in previous studies on fatalities involving khat. Although there is no conclusive evidence that khat caused death and its presence in the body does not necessarily imply that it did, it is expected that khat will cause death when consumed in large quantities [32]. As a result, it is critical to record and publish postmortem toxicology levels in khat fatalities.

The median blood concentrations of cathinone and cathine were 40 and 470 ng/mL, respectively, exceeding the median blood levels of cathinone and cathine in previously published forensic non-fatal road traffic accidents (median 33 and 129 ng/mL, respectively; N = 19) [33]. They conclude that chewing khat may severely impair driving ability. While the median urine concentrations of cathinone and cathine were 860 and 6240 ng/mL, respectively, in our study, they were less than the median levels of cathinone and cathine in the study mentioned (median 8000 and 38,600 ng/mL, respectively; N = 19). The concentrations of drugs in the antemortem and postmortem samples are not comparable because there are more uncertainties due to variable circumstances and incomplete information, and several other factors, such as postmortem redistribution and postmortem time interval, affect tissue concentrations in autopsy samples [34,35,36]. This may help to explain why drug tissue concentrations in postmortem versus antemortem samples differ. Additionally, cathinone is less stable and has a shorter elimination half-life (1.5 ± 0.8 h) than cathine (5.2 ± 3.4 h) [37,38]. As a result, cathine has a longer detectable period than cathinone. Nevertheless, urine concentrations indicate chemical exposure, but not acute toxicity. On other hand, cathinone blood concentrations ranged from 19 to 122 ng/mL in forensic postmortem cases [31]. These levels were lower than the wide range observed in our study (18–218 ng/mL), while cathine blood concentration was 1447 ng/mL in one forensic postmortem case, exceeding the range observed in our study (222–843 ng/mL). The average concentrations of cathinone and cathine in the brain, liver, and kidney were 69 and 682 ng/mL, 64 and 635 ng/mL, and 43 and 758 ng/mL, respectively. The concentrations of cathine were roughly 10 times higher than those of cathinone.

Cathine and cathinone should be included in all routine toxicological investigations, especially in homicidal cases, because the number of fatalities involving khat has already increased. This study reveals the number of deaths associated with khat use, both alone and in combination with other drugs. Which demonstrate that homicidal patterns were observed in a greater proportion of cases involving khat alone (77%) than cases involving khat in combination with other drugs (23%), and that suicidal pattern was observed in a greater proportion of cases involving khat in combination with other drugs (71.5%) than cases involving khat alone (28.5%).

Khat use has been associated with a higher risk of myocardial infarction [9,39,40,41]. It has been shown that khat induces tachycardia and hypertension, which may be increased at a higher dose, hence raising the risk of myocardial infarction [37,42,43]. Cathine and cathinone were the only substances detected in Case 26 of the current study, which showed ischemic heart disease with coronary partial occlusion.

Hepatotoxicity, on the other hand, has been observed in multiple studies, particularly among chronic khat chewers. Multilobular necrosis, canalicular cholestasis, an enlarged liver with decreased echogenicity, cirrhotic and portal fibrosis, jaundice with elevated liver enzymes, and severe liver injury with an elevated international normalized ratio were all characteristics of khat-related hepatic toxicity [44,45,46]. Hepatotoxicity was cured in two cases by discontinuing khat usage [10]. In the current study, cathine and cathinone were detected in the liver at average concentrations of 635 ± 132 and 64 ± 16 ng/mL, respectively. However, no evidence of liver toxicity was found.

Khat use has also been associated with mental disorders including anxiety, irritability, aggression, psychosis, paranoia, dysphoria, depression, insomnia, hallucinations, and delusions [47,48,49]. In addition, anxiety, trembling, lethargy, depression, and nightmares could be withdrawal symptoms [22,50,51]. On the other hand, khat intake may aggravate schizophrenia symptoms and diminish the therapeutic efficacy of antipsychotic drugs [52]. With both khat use and withdrawal condition, incidents of violence have been reported. In addition, anxiousness and poor decision-making have been observed following khat use [29,53,54]. Repeated oral administration of khat extract, according to Banjaw et al. (2006)’s report, makes experimental rats more aggressive [55]. Moreover, studies show that khat affects driving, social behavior, and work performance. It has been associated with homicide, notably in mentally ill people [11,48,53,56,57], and two reported homicides have been linked to khat consumption [56,58].

In the current study, homicide and suicide were the leading manners of death in fatalities involving khat alone and khat in combination with other drugs, respectively. In fatalities involving khat in combination with other drugs, particularly amphetamine, suicide was the most common manner of death. This study has limitations due to the small sample size and the fact that only non-natural cases were sent for toxicological analysis. In addition, it is essential to determine the relationship between khat consumption, violent crime, and fatalities. Therefore, a study is now underway to examine the role of khat in the toxicology of violent death over a ten-year period (2014 to 2023) and to establish reference ranges for cathinone and cathine in fatalities involving khat, which will represent only the fatal cases intoxicated with both cathinone and cathine.

In spite of the limitations described above, these findings suggest that fatalities involving khat are cause for concern, particularly among young people with a higher propensity for homicide and firearm injuries. Therefore, any health-based crime prevention strategy in Jazan has to consider khat use.

## 5. Conclusions

This study determined the average levels of cathinone and cathine in 30 cases of fatalities involving khat. This information may help forensic toxicologists and pathologists interpret toxicological investigations, but it should not be interpreted independently of other evidence, such as the death investigation and autopsy results. To create the body of literature necessary for more precise determinations of the role of khat in crimes and death investigations, additional research is required, particularly involving toxicological investigative and autopsy findings.

## Figures and Tables

**Table 1:** Postmortem toxicological analysis of cathinone in fatalities involving khat.

Cathinone Concentrations (ng/mL)						
Specimens	Brain	Liver	Kidney	Blood	Urine	Stomach ^1^
Number of samples	10	8	11	12	21	12
**Mean ± SEM**	69 ± 14.5	64 ± 16	43 ± 10	85 ± 26	1009 ± 204	Positive
**Median**	70	48	33	40	860	-
**10–90 Percentile**	25–126	30–138	13–99	18–218	105–2134	-

^1^ The stomach results indicate exposure, not acute toxicity.

**Table 2:** Postmortem toxicological analysis of cathine in fatalities involving khat.

Cathine Concentrations (ng/mL)						
Specimens	Brain	Liver	Kidney	Blood	Urine	Stomach ^1^
Number of samples	13	17	16	17	22	16
**Mean ± SEM**	682 ± 170	635 ± 132	758 ± 167	486 ± 80	12,616 ± 3279	Positive
**Median**	494	430	691	470	6240	-
**10–90 Percentile**	227–1471	162–1140	184–1530	222–843	1790–36,400	-

^1^ The stomach results indicate exposure, not acute toxicity.

**Table 3:** Summary of autopsy findings.

Case Number	Age (Year)	Cause of Death	Manner of Death	Detected Drugs
1	24	Blunt trauma to head and neck (skull fractures, brain hemorrhage)	Homicidal	Cathinone, Cathine
2	24	Firearm injury in head (skull fractures and brain lacerations)	Homicidal	Cathinone, Cathine
3	24	Firearm injuries in chest (lung lacerations and hemopneumothorax) and abdomen (hepatic, intestinal, and renal lacerations)	Homicidal	Cathinone, Cathine
4	25	Stab wounds in the chest (lung lacerations and hemopneumothorax)	Homicidal	Cathinone, Cathine
5	25	Firearm injuries in head (skull fractures and brain lacerations), neck, and chest (lung lacerations)	Homicidal	Cathinone, Cathine
6	35	Firearm injuries in head (skull fractures and brain lacerations), chest (lung lacerations), and abdomen (hepatic, intestinal, and renal lacerations)	Homicidal	Cathinone, Cathine
7	26	Firearm injury in neck (major blood vessels lacerations)	Homicidal	Cathinone, Cathine
8	30	Firearm injury in chest (lung lacerations and hemopneumothorax)	Homicidal	Cathinone, Cathine
9	47	Stab wounds in the chest (lung and heart lacerations)	Homicidal	Cathinone, Cathine
10	45	Firearm injuries in chest (lung and heart lacerations) and abdomen (hepatic and renal lacerations)	Homicidal	Cathine
11	61	Firearm injuries head (skull fractures and brain lacerations) and neck	Homicidal	Cathine
12	23	Firearm injury in head (skull fractures and brain lacerations)	Homicidal	Cathine, Amphetamine
13	25	Firearm injury in head (skull fractures and brain lacerations)	Homicidal	Cathine, Amphetamine
14	23	Suicidal hanging	Suicidal	Cathinone, Cathine
15	40	Suicidal hanging	Suicidal	Cathinone, Cathine
16	23	Suicidal hanging	Suicidal	Cathinone, Cathine, Olanzapine
17	32	Suicidal hanging	Suicidal	Cathinone, Cathine, Olanzapine
18	28	Suicidal hanging	Suicidal	Cathinone, Cathine, Amphetamine
19	38	Suicidal hanging	Suicidal	Cathinone, Cathine, Amphetamine
20	25	Suicidal hanging	Suicidal	Cathinone, Cathine, Amphetamine, Ethanol, THC
21	35	Road traffic accident (skull fractures, brain injury, thoracic cage fractures)	Accidental	Cathinone, Cathine
22	29	Road traffic accident (skull fractures, brain injury, thoracic cage fractures)	Accidental	Cathinone, Cathine
23	43	Head injury (skull fracture and extradural hemorrhage)	Accidental	Cathinone, Cathine
24	25	Choking	Accidental	Cathinone, Cathine, Amphetamine
25	26	Amphetamine poisoning	Accidental	Cathinone, Cathine, Amphetamine, Dextromethorphan, Diphenhydramine
26	42	Ischemic heart disease (partial occlusion in coronaries)	Natural	Cathinone, Cathine
27	29	Brain tumor (intraventicular benign tumor)	Natural	Cathinone, Cathine, Amphetamine
28	20	Unknown	Undetermined	Cathinone, Cathine
29	28	Unknown	Undetermined	Cathinone, Cathine
30	42	Carboxy-hemoglobin poising	Undetermined	Cathine, Amphetamine

## Data Availability

All relevant details are included in the article.
